# *Cortinarius pakistanicus* and *C. pseudotorvus*: two new species in oak forests in the Pakistan Himalayas

**DOI:** 10.3897/mycokeys.74.49734

**Published:** 2020-10-30

**Authors:** Arooj Naseer, Isaac Garrido-Benavent, Junaid Khan, Josep Ballarà, Rafael Mahiques, Abdul Nasir Khalid, Hassan Sher

**Affiliations:** 1 Department of Botany, University of the Punjab, Lahore, Pakistan University of the Punjab Lahore Pakistan; 2 Department of Biogeochemistry and Microbial Ecology, National Museum of Natural Sciences (CSIC), Madrid E-28006, Spain National Museum of Natural Sciences Madrid Spain; 3 University of Swat, Swat, Pakistan University of Swat Swat Pakistan; 4 Tossalet de les Forques 44, Berga E-08600, Spain Unaffiliated Berga Spain; 5 Doctor Climent 26, Quatretonda E-46837, Spain Unaffiliated Quatretonda Spain

**Keywords:** Biodiversity, ectomycorrhizal fungi, ITS, phylogeny, systematics, taxonomy

## Abstract

The genus of basidiomycetous fungi *Cortinarius* occurs worldwide, from subtropical to boreal latitudes. Although molecular systematics has triggered the study of these fungi in the Americas and Europe in the last two decades, there is still limited research on its diversity in large portions of the planet, such as the high mountain ranges of Asia. Several collections of *Cortinarius* were made during mycological field trips conducted between 2014 and 2018 in pure oak forests in the Pakistan Himalayas. An integrative framework combining morphological and phylogenetic data was employed for their study. As a result, the two species *C. pakistanicus* and *C. pseudotorvus* are here described as new to science. Detailed macro- and micro-morphological descriptions, including SEM images of spores, and a molecular phylogenetic reconstruction based on nrITS sequence data are provided and used to discriminate the new species from morphologically and phylogenetically close taxa. Whereas our phylogenetic tree inference gave unequivocal support for the inclusion of *C. pseudotorvus* within C. sect. Telamonia, the assignment of *C. pakistanicus* to any known sections remained elusive. These species likely establish ectomycorrhizal associations with trees in the genus *Quercus*, making this type of forest in the Pakistan Himalayas a promising focus for future research on the diversity of *Cortinarius*.

## Introduction

*Cortinarius* (Pers.) Gray (*Cortinariaceae*) is a relatively well known mushroom-forming genus of basidiomycetous fungi characterized by the fugacious veil forming a fine cobweb (“cortina”) between the stipe and pileus margin and by the production of ornamented, cinnamon brown to fulvous basidiospores (Kirk et al. 2008; Niskanen 2008). It is one of the most species rich, abundant and widespread ectomycorrhizal genera in *Agaricales* (Geml et al. 2012; Nouhra et al. 2013; Nuske et al. 2019), encompassing ca. 3000 species worldwide (Niskanen et al. 2018). Whereas the distributional ranges for many species are known to be rather restricted (e.g. Ballarà et al. 2017; Soop et al. 2019), other species occur across several regions and continents (Dima et al. 2014; Harrower et al. 2015; Liimatainen et al. 2017). Furthermore, evidence gained in recent years due to the use of high-throughput sequencing techniques has shown that *Cortinarius* establishes ectomycorrhizal associations with a myriad of plant hosts, including not only members in the important families *Fagaceae*, *Betulaceae*, *Pinaceae*, *Salicaceae* and *Cistaceae*, but also some herbaceous plants in the *Cyperaceae* and *Polygonaceae* (Geml et al. 2012; Horton et al. 2017; Pérez-Izquierdo et al. 2017; Nuske et al. 2019). However, the vast majority of systematic studies on *Cortinarius* have been conducted in Europe and the Americas, and more recently in Oceania (Australia, Tasmania and New Zealand), and therefore little is known about its diversity and range of plant hosts in other areas of the planet. In particular, we know virtually nothing about the species of *Cortinarius* growing in forests in the high mountain ranges north of Pakistan, which belong to the Himalayas.

Taxonomic studies on *Cortinarius* throughout the whole of Pakistan are in fact scant. Ahmad et al. (1997) reported *C. bulliardii* (Pers.) Fr., *C. cinnamomeus* (L.) Gray, *C. hinnuleus* Fr., and *C. phoeniceus* (Vent.) Maire from moist temperate forests of *Abies*, *Cedrus*, *Picea* and *Pinus* in Khanspur, Shogran, Dungagali and adjoining areas of the Pakistan Himalayas. After more than 20 years, Saba et al. (2017) described *C. longistipitatus* Saba, S. Jabeen, Khalid & Dima as a new species in C. subgenus Telamonia sect. Cinnabarini based on phylogenetic data. This species was collected in mixed *Cedrus deodara* and *Pinus wallichiana* forests in the Hazara and Malakand divisions. Recently, *C. brunneocarpus* Razaq & Khalid has been described from Khanspur growing in a mixed *P. wallichiana* and *Abies pindrow* forest, and the phylogenetic analysis found it in C. section Hinnulei (Song et al. 2019). In the same work, an additional species, *C. lilacinoarmillatus* Semwal & Dima, was described from the Indian Himalayas. All in all, further research on the diversity of this genus in the whole region is required. The aim of the present work is to provide new insights about the diversity of *Cortinarius* species associated with oaks in the Pakistan Himalayas.

**Figure 1.**
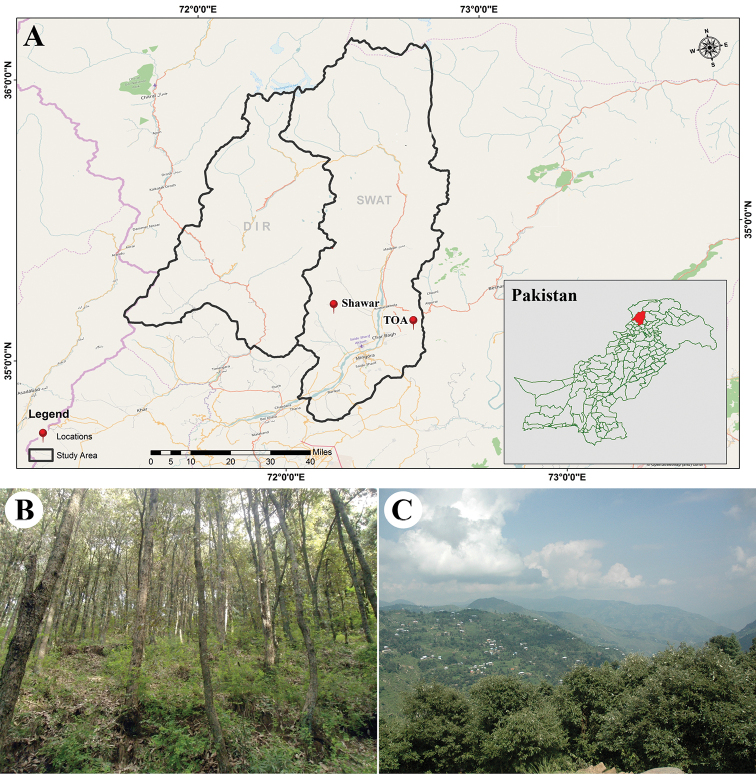
**A** Map of the two surveyed localities in the Swat District (Pakistan) **B** forest of *Quercus oblongata* in Shawar Valley **C***Quercus oblongata* trees in the Alpuri forests (Toa). Photo: A. Naseer.

Several collections of *Cortinarius* were sampled and preliminarily assigned to C. subgenus Telamonia (Fr.) Trog. during a 2014–2018 exploration campaign to oak forests from the Khyber Pakhtunkhwa province (Swat District, Pakistan, Fig. 1). This subgenus initially comprised a very large number of species, but thanks to the advent of molecular techniques, we now know that it is polyphyletic (Høiland and Holst-Jensen 2000; Niskanen et al. 2013a; Soop et al. 2019). Here, we combine data of the nuclear ribosomal internal transcribed spacer (nrITS) region as well as morphology and habitat to propose two novel *Cortinarius* species for science and study their affinities to any known sections within this genus.

## Methods

### Morphological and anatomical studies

Basidiomata were collected following Lodge et al. (2014) and photographed in their natural habitats using a Nikon D70S camera. Morphological characters were recorded from fresh specimens. Colors were designated with reference to mColorMeter application (Yanmei He, Mac App Store). Collections of the newly described species were deposited in the Herbarium of the Department of Botany, University of the Punjab, Lahore, Pakistan (acronym LAH). Microscopic characters are based on freehand sections from fresh and dried specimens mounted in 5% (w/v) aqueous Potassium Hydroxide (KOH) solution. Tissues from lamellae and pileipellis were mounted in phloxine (1%) for increasing contrast, and examined using a Meiji Techno MX4300H compound microscope. A total of 30 basidiospores, basidia, cystidia and hyphae from pilei were measured from each collection. For basidiospores, the abbreviation “*n/m/p*” indicates *n* basidiospores measured from *m* fruit bodies of *p* collections. Dimensions for basidiospores are given using length × width (L × W), and extreme values are given in parentheses. The range contains a minimum of 90% of the values. Measurements include the arithmetic mean of spore length and width.

### Laboratory procedures and sequence alignment and phylogenetic analyses

Genomic DNA was extracted from portions of lamellae following a modified CTAB extraction method (Bruns 1995). Primers used for amplification of the nrITS marker were ITS1F (Gardes and Bruns 1993) and ITS4 (White et al. 1990). Polymerase chain reactions (PCR) were performed in a total volume of 25 μL and consisted of an initial 4 minutes denaturation step at 94 °C, 40 cycles of 1 minute at 94 °C, 1 min at 55 °C, 1 min at 72 °C, and a final extension step of 10 minutes at 72 °C. Visualization of PCR products on a 1.5% agarose electrophoretic gel was done staining with SYBR Green. Successful amplicons were purified by enzymatic purification using Exonuclease I and Shrimp Alkaline Phosphatase enzymes (Werle et al. 1994). Bidirectional sequencing of purified products was done by Macrogen (Republic of Korea). Chromatograms were checked and assembled using SeqmanII v.5.07 (Dnastar Inc.).

The online tool BLAST (Altschul et al. 1997) and the databases GenBank (http://www.ncbi.nlm.nih.gov/) and UNITE (Nilsson et al. 2018) were used to check for possible PCR-product contamination and to identify and retrieve available, highly similar *Cortinarius* nrITS sequences to the four newly produced sequences (Table 1). Eighty-one sequences were downloaded, of which 52 corresponded to the sequence of the species’ type material. To provide a detailed view of *Cortinarius* phylodiversity, representative species of several sections were included: *C.*sect. Anomali (*Ano*, 2), *C.*sect. Biveli (*Biv*, 2), *C.*sect. Boulderenses (*Bou*, 2), *C.*sect. Bovini (*Bov*, 3), *C.*sect Brunnei (*Bru*, 2), *C.*sect. Castanei (*Cas*, 2), *C.*sect. Cinnabarini (*Cin*, 2), *C.*sect. Colymbadini (*Col*, 2), *C.*sect. Crassispori (*Cra*, 2), *C.*sect. Disjungendi (*Dis*, 2), *C.*sect. Duracini (*Dur*, 2), *C.*sect. Firmiores (*Fir*, 7), *C.*sect. Hinnulei (*Hin*, 4), *C.*sect. Hydrocybe (*Hyd*, 4), *C.*sect. Illumini (*Ill*, 2), *C.*sect. Incrustati (*Inc*, 1), *C.*sect. Infracti (*Inf*, 2), *C.*sect. Laeti (*Lae*, 2), *C.*sect. Malachii (*Mal*, 2), *C.*sect. Obtusi (*Obt*, 2), *C.*sect. Paleacei (*Pal*, 2), *C.*sect. Parvuli (*Par*, 2), the “*C. puellaris* group” (*Pue*, 3), *C.*sect. Safranopedes (*Saf*, 2), *C.*sect. Saniosi (*San*, 1), *C.*sect. Saturnini (*Sat*, 2), *C.*sect. Sciophylli (*Sci*, 2), *C.*sect. Telamonia s. str. (*Tel*, 8 spp.), *C.*sect. Uracei (*Ura*, 2), and *C.*sect. Urbici (*Urb*, 2). Two taxa within *C.* sect. Callochroi (Cal) were selected as outgroup: *C. calochrous* (Pers.) Gray and *C. barbarorum* Bidaud, Moënne-Locc. & Reumaux.

**Table 1.** Specimens included in phylogenetic analyses. Sequences produced in this study are highlighted in bold. Country codes follow www.country-code.cl/es/.

Species	Voucher	Country	GenBank and UNITE accessions (*nrITS*)
* Cortinarius acutus*	OS576	NO	KC842420
* C. alboviolaceus*	1734	IT	JF907875
* C. anomalus*	S: CFP1154 (Type)	SE	KX302224
* C. barbarorum*	TF2004-030	SE	DQ663237
* C. biformis*	SMIA42	CA	FJ039574
* C. bivelus*	IK-00518	PL	KX355542
* C. bovinus*	H: IK04-038 (Type)	FI	NR_120189
* C. boulderensis*	MICH: 10323 (Type)	US	NR_121207
* C. brunneocarpus*	LAH 240810 (Type)	PK	NR_166355
* C. brunneovernus*	WTU: JF Ammirati 13331 (Type)	US	NR_131826
* C. brunneus*	CFP587 (Type)	SE	DQ117927
* C. calochrous*	TF2001-113	SE	DQ663250
* C. caninus*	S: CFP627 (Type)	SE	KX302250
* C. chrysomallus* (= *C. saniosus*)	LY69_217 (Type)	FR	DQ102670
* C. cinnabarinus*	S: F248436 (Type)	SE	NR_120163
* C. coccineus*	GK: 435745 (Type)	FR	JX114945
* C. colymbadinus*	S: F248443 (Type)	SE	NR_131819
* C. confirmatus*	PC: R Henry 3195 (Type)	FR	KX964438
* C. crassisporus*	H: IK95-1085 (Type)	SE	NR_131882
* C. decipiens*	PML 366 (Type)	FR	FN428988
C. decipiens var. hoffmannii (= *C. casimiri*)	PML 559 (Type)	FR	FN429015
* C. denigrates*	WTU: M Beug 02MWB043014 (Type)	US	NR_153056
* C. disjungendus*	H: PA Karsten 4370 (Type)	FI	KP013190
* C. duboisensis*	WTU: J.F. Ammirati 13311 (Type)	US	NR_153057
* C. duracinus*	G: PML 349 (Type)	FR	KX964582
* C. flexipes*	MC01-551	DK	AJ889971
* C. fragantissimus*	WTU: M Beug 10MWB111913 (Type)	US	NR_153058
* C. fructuodorus*	H: 7001104 (Type)	US	NR_131827
* C. fulvescens*	TN04-935 (Type)	FI	NR_153077
* C. fuscescens*	H: 6001898 (Type)	FI	NR_131879
* C. gallurae*	CONS 00076 (Type)	IT	FN428979
* C. helobius*	HLCFP542	n/a	DQ102686
* C. hinnuleoarmillatus*	G: 00052098 (Type)	FR	NR_131790
* C. hinnuleus*	TUB 011512	DE	AY669665
* C. illuminus*	S: F44877 (Type)	SE	KP866156
* C. impennoides*	O: TE Brandrud TEB 281-09	NO	KT591607
* C. infractus*	TUB 011441	DE	AY174781
* C. infractiflavus*	SMI286	CA	FJ039612
* C. intempestivus*	PC: PML 1157 (Type)	FR	KX831120
* C. iunii*	J Ballarà JB-6989/10 (Type)	ES	MF000335
* C. laetus*	F15817	CA	FJ157034
* C. lilacinoarmillatus*	CAL: KCS2428 (Type)	IN	NR_166356
* C. malachius*	IK98-1298	FI	JX407332
* C. microglobisporus*	IB 20110123 (Type)	IT	NR_153027
* C. millaresensis*	J Ballarà JB-7369/11 (Type)	ES	KU953933
* C. minusculus*	H: TN12-032 (Type)	FI	MK211177
C. aff. multicolor	UBC: F17146 OC74	CA	GQ159889
* C. murinascens*	H: IK 08-958 (Type)	FI	KP165570
* C. neofallax*	PC: PML 1158 (Type)	FR	KF048129
* C. neofurvolaesus*	S: CFP1438 (Type)	SE	NR131789
* C. niveotraganus*	TN04-014a	FI	KM273104
* C. nolaneiformis*	PRM: J Velenovsky 857042 (Type)	CZ	NR_131833
* C. obtusus*	OS577	NO	KC842421
* C. olididisjungendus*	H: TN07-191 (Type)	CA	KM273091
* C. ortovernus*	JB-6048-08 (Type)	ES	KX964566
* C. oxytoneus*	PC: R Henry 931 (Type)	FR	KX964567
*** C. pakistanicus***	**AST332, LAH36366 (Type)**	**PK**	**MN864283**
*** C. pakistanicus***	**ASSW58, LAH35227**	**PK**	**MN864282**
* C. persimilis*	PC: GE 16.029 (Type)	FR	MH485205
* C. pseudofallax*	PC: 0124963 (Type)	FR	NR_131831
*** C. pseudotorvus***	**AST20, LAH35257 (Type)**	**PK**	**MN864285**
*** C. pseudotorvus***	**MJ-15103, LAH36368**	**PK**	**MN864286**
* C. puellaris*	O: TE Brandrud TEB 431-14 (Type)	NO	KT591581
* C. quarciticus*	H: CFP765 (Type)	SE	UDB000748
* C. roseocastaneus*	H:_6001997 (Type)	FI	NR_131866
* C. rubrovioleipes*	H: IK04-031 (Type)	FI	DQ497191
* C. saturninoides*	X Carteret 2013-144	FR	KX964573
* C. saturninus*	S: H Lindström CFP514 (Type)	SE	KX964584
* C. sciophylloides*	PC: A Bidaud 99-10-254 (Type)	FR	KX964576
* C. suberi*	IK95-349	FI	HQ845172
* C. subpuellaris*	O: TE Brandrud TEB562-15 (Type)	NO	KX831129
* C. subscotoides*	H: TN12-010 (Type)	FI	MK211175
* C. subserratissimus*	H: IK11-017 (Type)	SE	KP165552
* C. subturibulosus*	MES 3779	ES	FN428987
* C. torvus*	TUB 011515	DE	AY669668
* C. torvus*	TF01-035	DK	AJ889977
* C. torvus*	MIN: DJM1489	US	KY964778
* C. turgidoides*	PML35	FR	MH784724
* C. turgidus*	AB01-09-69	FR	MH784708
* C. umbrinobellus*	H: 7018158 (Type)	SE	NR_131874
* C. uraceisporus*	H: 6001791 (Type)	FI	NR_131878
* C. uraceonemoralis*	H: TN04-1116 (Type)	IT	KJ206515
* C. uraceus*	H: TN04-872 (Type)	FI	KJ206522
* C. urbicus*	H Lindström CFP486	SE	UDB000743
* C. venustus*	F16350	CA	FJ039571

MAFFT v.7.308 (Katoh et al. 2002; Katoh and Standley 2013) was used to build a multiple sequence alignment (MSA). The following parameter options were selected: the FFT-NS-I x1000 algorithm, the 200PAM / k = 2 scoring matrix, a gap open penalty of 1.5 and an offset value of 0.123. Manual editing of the resulting alignment was carried out in Geneious v.11.0.5 and consisted of trimming alignment ends of longer sequences that included part of the 18S–28S ribosomal subunits, and replacing gaps at the ends of shorter sequences with N (IUPAC bases representing any base). To assess the effect of keeping ambiguously aligned regions in the dataset, two analyses were conducted: one using the original MSA as input data, and the other using an MSA processed in GBlocks v.0.91b (Castresana 2000). This software allows for automatically removing these conflicting regions in the alignment and, to do this, the least stringent parameters were selected but allowing gaps in 50% of the sequences. Phylogenetic analyses were conducted under a Maximum Likelihood (ML) and Bayesian Inference (BI) perspectives, and used either the original MSA as well as the one trimmed with GBlocks. The ML phylogeny was inferred with the online version of RAxML-HPC2 hosted at the CIPRES Science Gateway (Miller et al. 2010; Stamatakis 2006; Stamatakis et al. 2008). Nodal support was evaluated with 1000 bootstrap pseudoreplicates. Then, the MrBayes analyses were conducted with two parallel, simultaneous four-chain runs executed over 5 × 10^7^ generations starting with a random tree, and sampling after every 500^th^ step. The first 25% of data were discarded as burn-in, and the 50% majority-rule consensus tree and corresponding posterior probabilities were calculated from the remaining trees. Optimal substitution models for the two partitions within the nrITS (ITS1+2, 5.8S) used in the above analyses were inferred with PartitionFinder v.1.1.1 (Lanfear et al. 2012) considering a model with linked branch lengths and the Bayesian Information Criterion (BIC). This analysis favored the GTR+Γ model for the two nrITS partitions in the RAxML analyses, whereas in the MrBayes analyses, the GTR+Γ and K80 models were selected for ITS1+2 and 5.8S, respectively. Average standard deviation of split frequencies (ASDSF) values below 0.005 and potential scale reduction factor (PSRF) values approaching 1.00 were considered as indicators of chain convergence in the Bayesian analyses. As for tree nodal support, nodes showing Bootstrap support (BS) values equal or higher than 70% (RAxML analyses) and Bayesian posterior probabilities (PP) equal or higher than 0.95 (MrBayes analyses) were regarded as significantly supported. Phylogenetic trees were visualized in FigTree v.1.4 (available at http://tree.bio.ed.ac.uk/software/tracer/) and Adobe Illustrator CS5 was used for artwork.

## Results

### Molecular phylogenetic analyses

**Figure 2.**
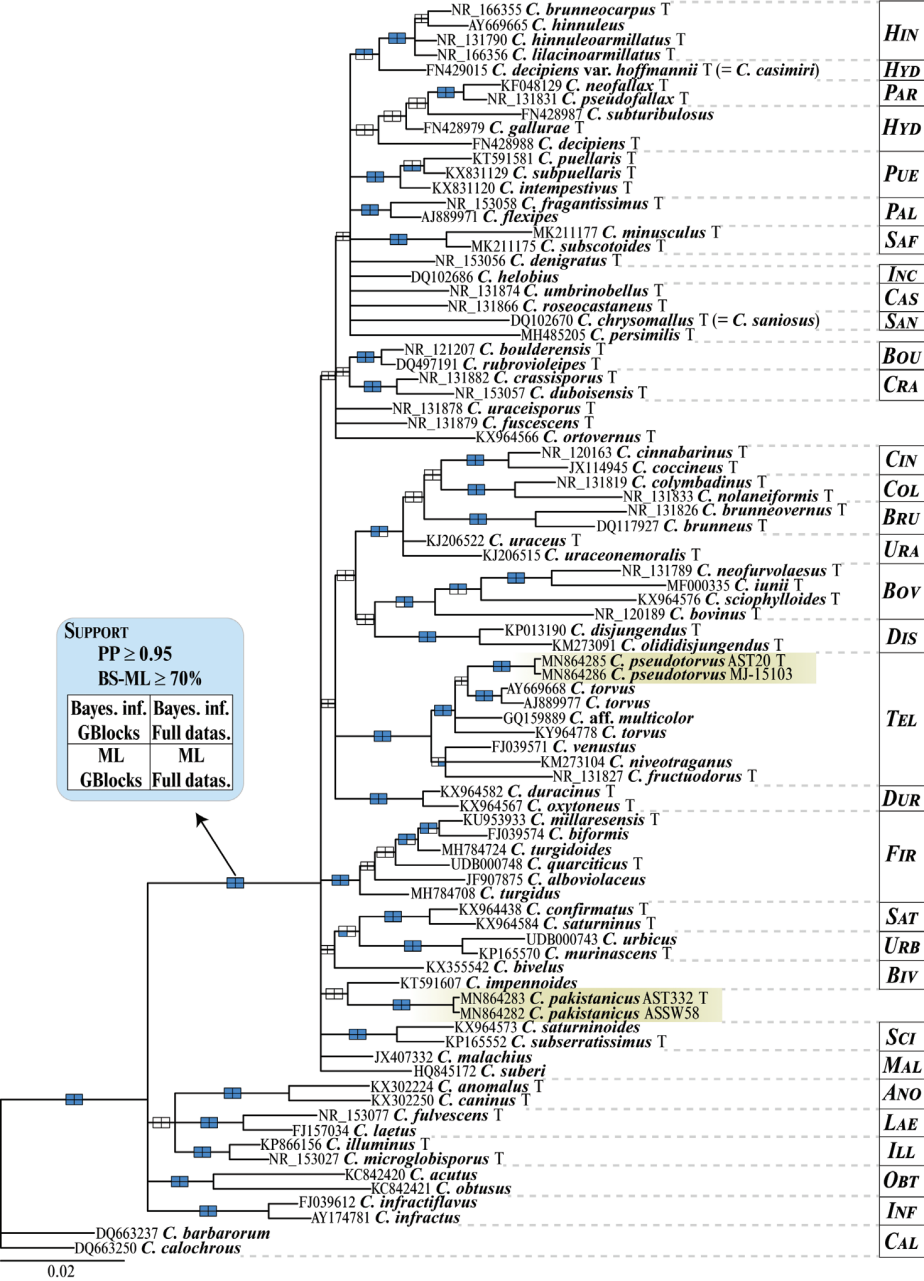
Phylogram depicting the evolutionary relationships of the new *Cortinarius* species and their relatives. The represented topology was obtained under a Bayesian framework using MrBayes and the GBlocks-trimmed dataset. For each terminal, the GenBank nrITS accession number, species name and an indication when they are type material (T) are given. Posterior Probabilities (PP, Bayesian analyses) and Bootstrap support (BS, RAxML analyses) are represented on branches leading to nodes. Blue-filled rectangles indicate nodal support for any of the four analyses performed in this study. *Cortinarius* sections in which the considered species belong are showed in the right column (see abbreviations list in section Material and methods).

The original MSA produced with MAFFT was 701 base pairs in length (347 variable and 105 singleton sites), whereas the GBlocks-trimmed MSA comprised 494 positions (70% of the original length with 216 variable and 60 singleton sites) distributed in 45 blocks (Table 2). The ML analysis in RAxML generated a phylogeny with Ln = -6706.607367 (original MSA) and Ln = -5097.173288 (GBlocks-trimmed). The MrBayes analyses reached an average standard deviation of split frequencies of 0.005 after 2.636 × 10^7^ (original MSA) and 1.2295 × 10^7^ (GBlocks-trimmed) generations. Average Estimated Sample Sizes (EESs) were well above 200 in both Bayesian analyses. Because the four inferred phylogenies (two ML, and two BI) were largely coherent, with no supported conflicts, the topology inferred with MrBayes using the GBlocks-trimmed MSA is presented in Fig. 2.

**Table 2.** Polymorphism statistics for the two versions of the nrITS dataset. In both cases, the number of species included was 52.

	Full *nrITS* dataset	GBlocks-trimmed *nrITS* dataset
Number of aligned sites (bp)	701	494
Partition ranges (bp)	*ITS1*+*ITS2*: 1–543	*ITS1*+*ITS2*: 1–336
	*5.8S*: 544–701	*5.8S*: 337–494
Singleton sites	105	60
Parsimony informative sites	235	156
Number of polymorphic sites	347	216
Conserved sites	328	278

In general, analyses retrieved BS > 70% and PP > 0.95 for clades that represented several *Cortinarius* sections (Fig. 2). All analyses supported a clade containing *C.* sections *Hinnulei*, *Hydrocybe*, *Parvuli*, *Paleacei*, *Safranopedes*, *Incrustati*, *Castanei*, *Saniosi*, *Boulderenses*, *Crassispori*, *Cinnabarini*, *Colymbadini*, *Brunnei*, *Uracei*, *Bovini*, *Disjungendi*, *Telamonia*, *Duracini*, *Firmiores*, *Saturnini*, *Urbici*, *Biveli*, *Sciophylli*, *Malachii* and the “*C. puellaris* group”. Among these, evolutionary relationships remained mostly opaque, as is often the case in phylogenetic analysis including only one molecular marker, and very divergent sequences among taxa (e.g. Brandrud et al. 2018; Garrido-Benavent et al. 2019). The two Pakistani collections AST20 and MJ-15103 were genetically identical. The closest relatives were two GenBank samples (AY669668, AJ889977) labelled as *C. torvus* (Fr.) Fr. that were collected in Europe (Germany and Denmark) although this relationship was not statistically supported. The number of divergent nucleotides between AST20, MJ-15103 and these two *C. torvus* samples were nine plus 5 indels. A third *C. torvus* sample (KY964778) and a sample labelled as C. aff. multicolor (GQ159889), both collected in North America, showed a higher number of nucleotide differences. Along with *C. venustus* P. Karst., *C. fructuodorus* Niskanen, Liimat. & Ammirati and *C. niveotraganus* Kytöv., Niskanen & Liimat., these samples formed a well-supported clade corresponding with C. sect. Telamonia. Therefore, these results demonstrate that this clade of cortinarii is widely distributed across several landmasses in the Northern Hemisphere.

The two Pakistani collections AST332 and SW58 were genetically identical as well, but showed no close relationship to any other *Cortinarius* sample found in current databases. The closest relatives in the phylogenetic reconstructions obtained in this study were members in *C.* sections *Biveli*, *Sciophylly*, *Urbici*, *Saturnini*, and *Firmiores* although no statistical support was found for the inferred relationships (Fig. 2). Sequences in these clades diverged from AST332 and SW58 by at least 20 nucleotides and a number of indel positions. Given the evidence collected in these phylogenetic analyses and morphological comparisons with non-sequenced telamonioid taxa found in literature, the two lineages of *Cortinarius* sampled in the Pakistan Himalayas are considered to represent new species for science and hence are described below.

### Taxonomy

#### Cortinarius pakistanicus

Taxon classificationFungiAgaricalesCortinariaceae

A. Naseer & A. N. Khalid sp. nov.

E5232507-7CDA-5F8B-95E7-B65E85CDE60B

MycoBank No: 833818

Figure 3

##### Diagnosis.

*Cortinarius pakistanicus* is an oak-associated species that forms small basidiomata, with campanulate to obtusely umbonate, slightly hygrophanous, dark reddish to brownish pilei with margins first incurved and with whitish veil traces; lamellae are fairly distant, first pale brown and later dark brown, with edges lighter and fimbriate; stipes slender, cylindrical to slightly fusiform, hollow, with a barely fibrillose surface, lilaceous towards the apex and whitish-tomentose towards the base, the remaining brown to dark brown with age and without annular traces; it produces amygdaliform, profusely and coarsely verrucose basidiospores measuring 8.6 × 5.5 µm, and shows cylindrical to narrowly utriform marginal cells.

##### Type.

Pakistan, Khyber Pakhtunkhwa province, Swat, Toa, Alpuri forests, 34°51'51.2"N, 72°39'48.0"E, 2800 m a.s.l., on soil under *Quercus oblongata*, leg. Arooj Naseer & Abdul Nasir Khalid, 1 August 2018, AST332 (LAH36366).

##### Etymology.

The epithet “*pakistanicus*” refers to Pakistan, where the species was collected.

##### Description.

*Basidiomata* small sized. ***Pilei*** campanulate to umbonate, 2 to 3.3 cm in diameter, with margins first incurved, and then involute when mature, slightly hygrophanous; cuticle dark reddish to brownish (7.6 YR 3.6/5.8) with lighter brown tinges (1.6Y 6.6/3) towards the margin, fibrillose, and margins fimbriate. ***Lamellae*** medium spaced to fairly distant, emarginate, serrate to undulate, broad, first pale brown and later dark brown (4.7 YR 1.8/2.7). ***Stipe*** clavate to cylindrical, straight to curved, 5–9 cm long, 0.5–1 cm thick, slightly tapering towards the apex, which is about 0.3–0.7 cm, hollow; surface longitudinally striate, lilaceous towards the apex, and brown (1.8Y 6.6/2.7) to dark brown (7.6 YR 3.6/5.8) with age, base whitish (7.2GY 8.4/0.7). ***Context*** of pileus and stipe the same color as the cuticle. ***Smell*** distinct, earth-like and ***taste*** not recorded.

***Basidiospores*** thin-walled, ellipsoid, [90/6/3] (7.8–) 7.9–9.4 (–9.8) × (4.7–) 4.9–6.3 (–6.2) μm in size, avl × avw = 8.62 × 5.56 μm, non-amyloid. ***Basidia*** clavate, 27.38 × 7.81 μm, 4-spored, clamped at the base, hyaline in 5% KOH. Scant cellular elements in lamellar pleura cylindrical to narrowly utriform 30–35 × 5–8 µm, clamped at the base. ***Pileipellis*** duplex; epicutis composed of individual hyphae 3–4 µm in diameter, clamped at septa, and with clavate to cylindrical terminal ends.

##### Ecology.

Gregarious; growing in mountainous pure oak forests of *Quercus oblongata* at an altitude greater than 2000 m a.s.l. The soil pH ranged between 6–8.4.

##### Additional material examined.

Pakistan, Khyber Pakhtunkhwa province, Swat, Toa, Alpuri forests, 34°51'51.2"N, 72°39'48.0"E, 2800 m a.s.l., on soil under *Quercus oblongata*, leg. Arooj Naseer & Abdul Nasir Khalid, 6 August 2016, AST132 (LAH36367); Swat, Matta, Shawar Valley, 34°58'59.8"N, 72°19'36.5"E, 2100 m a.s.l., solitary on ground under *Quercus oblongata*, 14 July 2015, Arooj Naseer & Abdul Nasir Khalid, ASSW58 (LAH35227).

**Figure 3.**
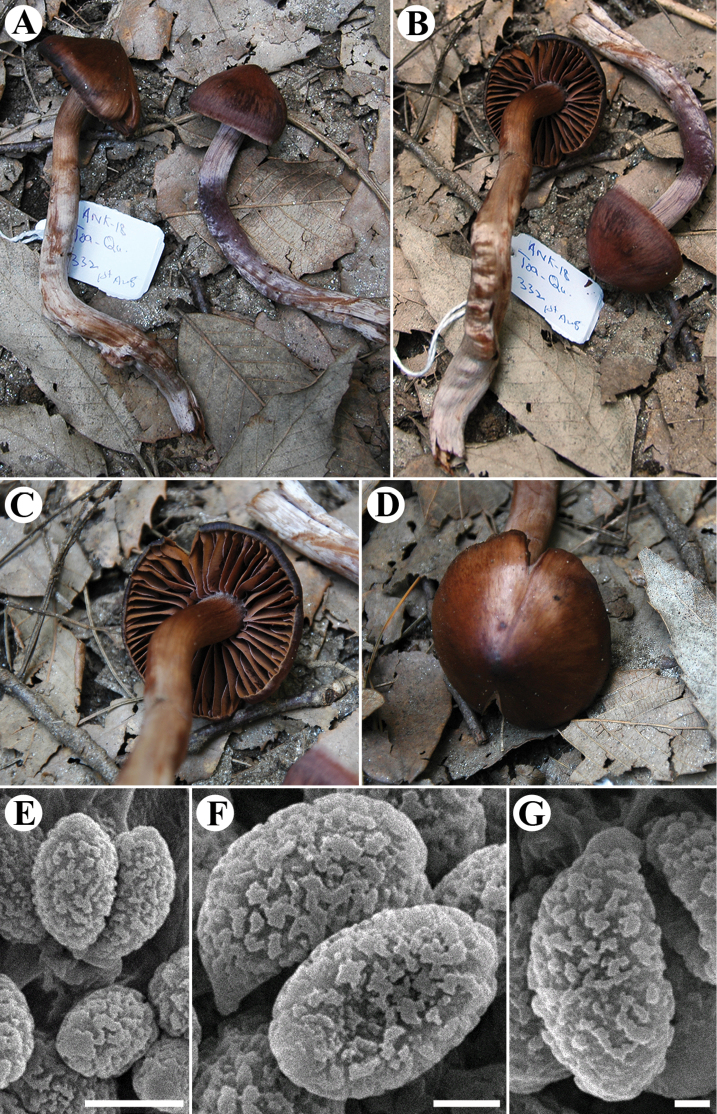
The new species *Cortinarius pakistanicus*, AST332 (LAH36366, holotype). **A–D** Basidiomata **E–G** Basidiospores observed with the SEM technique. Photo: A. Naseer (**A–D**).Scale bars: 5 µm (**E**), 2 µm (**F**), 1 µm (**G**).

#### Cortinarius pseudotorvus

Taxon classificationFungiAgaricalesCortinariaceae

A. Naseer, J. Khan & A. N. Khalid sp. nov.

80B517D9-0549-5785-9AB1-3AC25B9AA951

MycoBank No: 833817

Figure 4

##### Diagnosis.

*Cortinarius pseudotorvus* is an oak-associated species that differs from *C. torvus* by the smaller and slender basidiomata, and by the slightly more felty surface of pilei; it has broadly ellipsoid to sub-amygdaliform basidiospores (10.9 × 7.1 µm in average).

##### Type.

Pakistan, Khyber Pakhtunkhwa province, Swat, Toa, Alpuri forests, 34°51'51.2"N, 72°39'48.0"E, 2800 m a.s.l., on soil under *Quercus oblongata*, Arooj Naseer & Abdul Nasir Khalid, 15 July 2015, AST20 (holotype: LAH35257).

##### Etymology.

The epithet “*pseudotorvus*” indicates its morphological resemblance and close phylogenetic position to *Cortinarius torvus*.

##### Description.

*Basidiomata* small sized and slender. ***Pileus*** campanulate when young, becoming subumbonate and sometimes flat to plano-convex in mature stages, 15–30 mm in diameter, with margins deflexed, undulate, sometimes moderately to strongly striate; cuticle light brown (8.3YR 6.7/2.5), smooth to finely or even coarsely fibrillose (felty), with dark brown (4.7YR 2.6/5.5) fibrils radiating from the center. ***Lamellae*** adnate, broad, distant and relatively thick, with an evenly smooth margin, dark brown with age (4.1 YR 1.8/3.1); *lamellulae* present, regular. ***Stipe*** cylindrical, up to 56 mm long, 4–9 mm at apex and 1.1–1.3 cm thick at base, which is slightly bulbous, solid; surface brown (7.8YR 4.4/4.7) becoming whitish (3Y 7.2/1.5) in upper half and the base, finely fibrillose, the 3⁄4 of stipe covered with universal veil remnants when young, and partial veil present, whitish in young specimens and brownish when mature, forming in general a persistent annulus. ***Context*** of pileus and stipe of the same color as the cuticle. ***Smell*** indistinct and ***taste*** not recorded.

***Basidiospores*** broadly ellipsoid to sub-amygdaliform, [90/6/3] (9.2–) 9.9–11.6 (–12.5) × (6.1–) 6.7–7.7 (–8.1) µm, avl × avw= 10.9 × 7.1 µm, light yellowish brown to dark brown in 5% KOH, reddish brown in Melzer’s reagent, densely ornamented. ***Basidia*** clavate, 25–35 × 7–8 µm, 4-spored, clamped at the base, hyaline in 5% KOH, darker when stained in Congo red. Scant cellular elements in lamellar pleura cylindrical to narrowly utriform, 30–35 × 5–8 µm, and clamped at the base. ***Pileipellis*** duplex; epicutis composed of individual hyphae 3–4 µm in diameter, clamped at septa and with clavate to cylindrical terminal elements.

##### Ecology.

Gregarious; growing in either pure oak forests (*Quercus oblongata*) or mixed forests with oaks and pines (*Pinus wallichiana*) at an altitude greater than 2000 m a.s.l. The soil pH was around 8.4.

##### Additional material examined.

Pakistan, Khyber Pakhtunkhwa, District Swat, Malam Jabba valley, 34°50'57.6"N 72°33'15.7"E, on ground in mixed forests of oak and pines, Junaid Khan, 10 August 2018, MJ-15103 (LAH36368); Toa, Alpuri forests, 34°51'51.2"N 72°39'48.0"E, 2800 m a.s.l., on soil under *Quercus oblongata*, Arooj Naseer & Abdul Nasir Khalid, 15 July 2015, AST17 (LAH35256).

**Figure 4.**
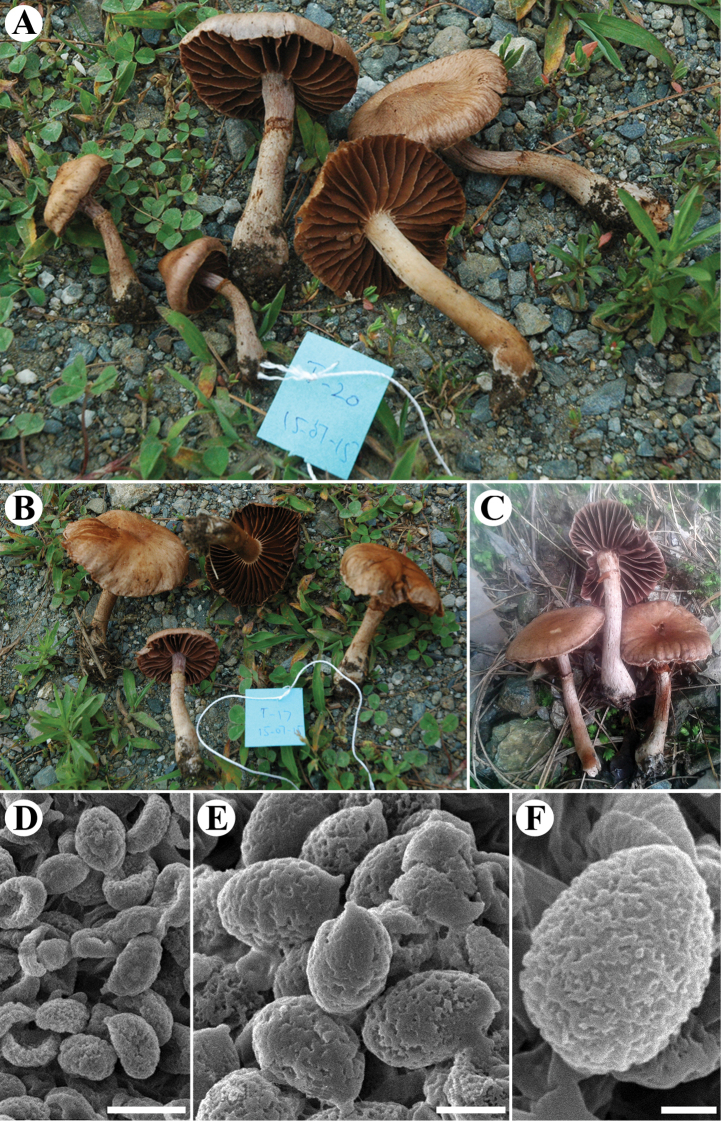
The new species *Cortinarius pseudotorvus*, AST20 (LAH35257, holotype). **A–C** Basidiomata **A, B** AST20 (holotype) **C** MJ-15103 (LAH36368) **D–F** Basidiospores of AST20 (holotype) observed with the SEM technique. Photo: A. Naseer (**A, B**), J. Khan (**C**). Scale bars: 10 µm (**D**), 5 µm (**E**), 2 µm (**F**).

## Discussion

In the present study, *Cortinarius pakistanicus* and *C. pseudotorvus* are described as new to science based on an integrative taxonomic approach. The brownish color with occasional lilaceous tinges displayed by basidiomata of these species, which are both of rather small size, suggested their inclusion within the complicated C. subgenus Telamonia s. lat. Although recent phylogenetic surveys have helped to better circumscribe it, telamonioid cortinarii are still poorly known and constitute one of the most taxonomically challenging groups within the genus (Niskanen et al. 2013b; Brandrud et al. 2015, Liimatainen et al. 2015; Garrido-Benavent et al. 2019).

Our phylogenetic inference showed that *C. pakistanicus* is not in any known sections. The closest taxa to this species were *C. bivelus* (Fr.) Fr. and *C. impennoides* Bidaud, Moënne-Locc. & Reumaux in C. sect. Biveli, which produce larger basidiomata that become pale ochraceous when dry, their spores are shorter and they usually grow in subalpine or boreal regions (Brandrud et al. 2015). In any case, phylogenetic relationships among these species were not supported by any of the four different phylogenetic analyses implemented in the present study. Furthermore, other species in C. sect. Biveli usually have abundant whitish veil remnants and lack any traces of lilaceous tinges in basidiomata which are otherwise shown by *C. pakistanicus* at least on the stipe surface. Young specimens of *C. pakistanicus* may resemble several species in C. sect. Hydrocybe due to overall morphology, pigmentation and the presence of traces of veil in the surface of pilei (Suárez-Santiago et al. 2009), although our reconstructed phylogenetic tree showed that members of this section are phylogenetically unrelated to the new species. The type species of this section is *C. decipiens* Fr., which usually shows grayish lamellae and stipe cortex when young, traits that were not noticed on our specimens. Besides, *C. decipiens* has had various interpretations in the literature and it probably forms a species complex in need of detailed morphological and phylogenetic study (e.g. Esteve-Raventós et al. 2014; Brandrud et al. 2015). *Cortinarius gallurae* D. Antonini, M. Antonini & Cons. can be larger in size, with flattened pilei ca. 60 mm showing abundant veil traces, and has shorter spores than the new species and associates with thermophilous *Quercus* spp. in the Mediterranean region (Consiglio et al. 2005). *Cortinarius casimiri* (Velen.) Huijsman displays a warmer pigmentation than *C. pakistanicus* and its spores are larger, ca. 12 × 7 µm. *Cortinarius subturibulosus* Kizlik & Trescol has a distinct smell like orange flower and associates with thermophilous *Quercus* spp. (Ortega and Mahiques 1995). On the other hand, mature and dry *C. pakistanicus* specimens with an umbonate pileus may bear a slight resemblance to species in C. sect. Hinnulei, but these normally lack lilaceous tinges, show more evident veil remnants on the stipes and their spores are more coarsely ornamented. *Cortinarius saniosus* (Fr.) Fr. in C. sect. Saniosi produces basidiomata with a similar size but these display warmer colors and a striking yellowish veil. Members in other sections like C. sect. Brunnei and *Uracei* commonly associate with coniferous trees and are characterized by producing slender or large basidiomata, with darker pigmentation than *C. pakistanicus*, sometimes necropigmented, and highly hygrophanous pilei.

On the other hand, the inferred nrITS phylogeny was unequivocal in including *C. pseudotorvus* within C. sect. Telamonia, together with several representatives of the section’s type species *C. torvus*. Both species share the habitat under deciduous trees, the fibrillose to felty and matte pileus cuticle, which is slightly more felty in the new species, and the relatively thick and distant lamellae (Brandrud et al. 1992: B13). In *C. torvus* lamellae are initially grayish, a character that was not noticed in our collections, and then they turn brownish and even darker with age as happens in *C. pseudotorvus*. Spore dimensions are similar in both taxa, despite verrucae in the new species’ spores seeming to be less prominent. Nevertheless, the main distinguishing character between *C. torvus* and *C. pseudotorvus* is that the former produces stouter and larger basidiomata. This is particularly true for *C. torvus* stipes, whose morphology is also more variable, from cylindrical (as in *C. pseudotorvus*) to fusiform and especially clavate. A further character that should be tested with new collections of *C. pseudotorvus* is the smell, which in *C. torvus* is described as persistently sweet and pleasant. There are other species within C. sect. Telamonia displaying such smell and with abundant veil remnants: *C. venustus* P. Karst., whose basidiomata are more vividly pigmented, with lilaceous to orangish tinges (Brandrud et al. 1994: C50); and the very hygrophanous *C. agathosmus*, with dark pilei when moist and with longer stipes (Brandrud et al. 1990: A05). In contrast to *C. pseudotorvus*, both species fructify in acidophilous, subalpine coniferous forests. Finally, *C. tigrinipes* Bergeron displays conspicuous bands or girdles on the stipe, which is not thickened at the base (as in *C. pseudotorvus*), and the spores are smaller (7.5) 8–10.5 × 5–6.5 µm (Bidaud et al. 1999: pl. 250, f. 410).

With the new data provided in the present study, the number of *Cortinarius* species for all of Pakistan increases to eight. In neighboring countries, such as India, the number of recorded species for this genus seems to be lower than 20 as well (Verma et al. 2019). All in all, these data suggest that our knowledge of the diversity of *Cortinarius* in high mountain areas in Asia and the Himalayan forests of Pakistan is still in its infancy. By combining morphological and molecular analyses, which has proven to be a straightforward approach to disentangle the diversity of other fungal groups in the region (Saba et al. 2015; Khan et al. 2017; Sarwar et al. 2018; Naseer et al. 2019), we expect to further improve our knowledge of the mycobiota in this area of the planet.

## Supplementary Material

XML Treatment for Cortinarius pakistanicus

XML Treatment for Cortinarius pseudotorvus
